# Between paternalism and illegality: a longitudinal analysis of the role and condition of manual scavengers in India

**DOI:** 10.1136/bmjgh-2022-008733

**Published:** 2022-07-22

**Authors:** Sakshi Saldanha, Claas Kirchhelle, Emily Webster, Samantha Vanderslott, Manjulika Vaz

**Affiliations:** 1 Health and Humanities, St John's Research Institute, St John's Medical College, Bangalore, India; 2 Department of History, University College Dublin, Dublin, Ireland; 3 School of History, University College Dublin, Dublin, Ireland; 4 Oxford Vaccine Group, University of Oxford, Oxford, UK

**Keywords:** COVID-19

## Abstract

Manual scavengers, or ‘Safai Karamcharis’, as they are known in India, are sanitation workers who manually clean human waste for a living and face considerable occupational health risks. They are subject to deep-seated, caste-based stigma associated with their perceived ‘caste impurity’ and lack of cleanliness, which result both in consistently dangerous substandard working conditions and lack of social mobility, with women facing greater hardships. The COVID-19 pandemic has further exacerbated their plight. Despite the considerable efforts of social advocates, organised movements and government institutions, reforms and criminalisation have produced mixed results and campaigners remain divided on whether banning manual scavenging is an effective solution. This article reviews the history of attempts to address scavenging in India. Starting in the colonial period and ending with the current government’s Swachh Bharat Mission, it highlights how attempts to deal with scavenging via quick-fix solutions like legal bans criminalising their employment, infrastructure upgrades or paternalistic interventions have either failed to resolve issues or exacerbated scavengers’ situation by pushing long-standing problems out of view. It argues that meaningful progress depends on abandoning top-down modes of decision-making, addressing the underlying sociocultural and infrastructural factors that perpetuate the ill health and social conditions of manual scavengers, collecting data on the true extent of scavenging, and investing in and providing political agency to communities themselves.

**Summary box**

- According to labour standards, Indian Manual Scavengers face considerable social stigma and occupational health risks.
- Laws banning manual scavenging have not eradicated manual scavenging in India.
- Skill upgradation schemes, sanitation infrastructure development and social welfare measures for manual scavengers have been attempted over the years by several governments in India.
- Despite various substantive reports, government and municipal agencies deny the existence and employment of manual scavenging.

## Introduction

Across Indian cultures and communities, sanitation work has been the responsibility of the lowest castes.^1^ The Hindi term ‘Safai Karamcharis/Karmacharis’ (cleaning worker or staff) is commonly used to refer to sanitation workers and sweepers. The term ‘Karamchari’ is also used in current legal lexica when referring to manual scavengers,^2^ who engage in manually cleaning, carrying, handling or disposing of human excreta in specifically insanitary conditions, such as open drains, pits or railway tracks.^3^ This dangerous work is overwhelmingly performed by people from Scheduled Castes, who face resulting serious health risks from faecally transmitted infectious disease, noxious chemicals, asphyxiation and injury, as well as substantial social discrimination due to the ‘polluting’ nature of the work.^4^ To address the demeaning nature of manual scavenging, its health risks and the social stigma experienced by scavenging communities, numerous Indian administrations have resorted to interventions ranging from upgrading sanitary infrastructures to passing paternalistic legislation designed to protect scavengers while perpetuating their status, to rendering the employment of manual scavengers illegal.

This paper assesses the impact of these reforms on Safai Karamcharis and their communities. Following an overview of the significant health costs communities incur, we conduct a historical survey of legal arrangements from ca. 1900 onwards. We draw on published literature in peer-reviewed journals in medicine, humanities and social sciences. We complement this review of published academic material by analysing newspaper reports, grey or non-academic literature (mostly from between 1990 and 2021), legal and policy documents, and detailed accounts of the experiences of the workers themselves to create a multiperspective analysis of the extent and evolution of Safai Karamcharis’ legal and social status.

Our findings highlight that shifting political priorities, technocratic optimism, paternalism (defined here as top-down restrictions on the freedoms of subordinate peoples under a governance structure that simultaneously provides limited protection and support), and neoliberal attempts to serve market-based supply and demand of scavenging labour, have repeatedly prioritised quick-fix legal and technological solutions over structural interventions. We argue that improvement of Safai Karamcharis’ situation depends not only on upgrading sanitary infrastructure, but also on empowering communities by collecting data on the true extent of scavenging and ending their economic, sociocultural and political obfuscation.

## The occupational and community health risks of scavenging

Manual scavenging is an extremely dangerous occupation. According to the Press Trust of India, 115 manual scavengers died while cleaning sewers and septic tanks in 2019.^5^ This figure is likely an underestimate due to the illegal nature of scavenging, and the fact that it only captures on-the-job deaths due to asphyxiation or drowning in sewers and septic tanks, excluding deaths that take place offsite due to infections and respiratory or cardiovascular incidents, among many other causes.^6 7^ According to social activists from the state of Maharashtra, the average annual death rate among Safai Karamcharis was estimated to be 9 per 1000 in 2016, nearly a third higher than the 6.7 per 1000 found among the general population. Deaths also appear to occur at a younger age among Safai Karamcharis, with their mean life expectancy at 58 years compared with 68.3 years in the general population.^8^

Gender plays a significant role in the degree of occupational health risk that scavengers face. Women reportedly comprise over 95% of all manual scavengers in India and tend to be charged with cleaning human excreta from dry latrines as it is the lowest paid job.^9 10^ Although they also face stigmatisation and prejudice, rehabilitation and occupation mobility are comparatively easier to access for male scavengers as they have fewer ‘household responsibilities’ or ‘demands of childcare’.^1^ In addition, men often perform the more skilled work of waste removal involving machinery and transport.^1^ Working without personal protective equipment (PPE) and legal protection, Karamcharis describe various afflictions resulting from chronic exposure to pathogens and the toxic, carcinogenic and teratogenic pollutants commonly found in sewage. Problems include aggressive itchy patches, rashes, boils and other skin infections. Physiological damage also results from carrying heavy, leaky baskets on their head, including hair loss, postural problems and miscarriages.^11^ Reduced general immunity, indicated by falling sick constantly, was also stated as common.^11^

Occupation-related poor nutrition may exacerbate health problems. Scavengers also report an aversion to eating *dal* (an important source of protein in India) and other foods after handling faeces all day. Despite thorough washing of hands, a female scavenger stated that her own hands and nails repulsed her, claiming “Cleaning the shit of these people is bad enough. I don’t want to put that in my mouth.”^12^ Mental health and addiction problems resulting from precarious and illegal employment, and social stigma are also reportedly common among these communities.^13^

Scavenging communities face additional secondary health risks by directly or indirectly exposing family members to pathogens via touch or food preparation.^11^ Exposure is increased due to a lack of appropriate cleaning facilities. In a survey conducted during the 2020 COVID-19 lockdown, the non-governmental organisation (NGO) WaterAid found that 40% of sanitation workers, who were pressured by the national government to remain on the job, lacked handwashing facilities at their workplace. Similarly, the informal and often crowded settlements that many workers live in often lack access to appropriate water and sanitation infrastructure as well.^14^ Meanwhile, potential long-term intergenerational impacts of mothers’ exposure to various chemical substances (including endocrine disruptors like endosulfan or ‘forever chemicals’ like high performance polyamides/ Polyphthalamide or PPAs) on unborn children remain understudied.^15^

## A sociocultural history of scavenging

The disproportionate mortality and morbidity faced by scavenging communities has deep roots in Indian society and history. Understanding the extent of these roots as well as political path dependencies created by previous administrations’ attempts to address scavengers’ plight is important when it comes to developing more robust interventions moving forward. Looking back at the past century of reform efforts, what emerges is a complicated history in which a long-term mix of socioeconomic, cultural and biological precarity has only ever been partially addressed by regulatory interventions.

The caste system in India has long divided communities into a distinct social hierarchy based on concepts of cleanliness embedded in Hindu philosophy.^16^ While commonly used in India today, the word ‘caste’—derived from the Latin ‘castus’ meaning ‘chaste’—was initially used by the Portuguese in the mid-16th century to denote Hindu societal stratification^17^ into four primary groups with distinct social privileges and duties.^18^ Concepts of purity and pollution play an important role in these divisions.^19^ Bodily fluid, faecal matter and carcasses are deemed pollutants, both in terms of cleanliness and holiness.^20^ While occupation is no longer as rigidly assigned as it was centuries ago, workers tasked with cleaning and disposing pollutants are still predominantly recruited from the lowest castes and continue to be labelled as polluted themselves, both physically and spiritually. Estimates suggest that today roughly 95% of manual scavengers belong to the Scheduled Castes.^4^ Historically, this caste-based division of labour has been justified with a doctrine of responsibility to the community (*chaturvarna*), which sees the role of Karamcharis as providing an essential community service by taking up ‘polluting’ work and allowing members of higher castes to maintain their purity. This concept of service includes aspects of social and spatial segregation such as expectations for Karamcharis to mark themselves, announce their presence and physically exclude themselves from areas designated for ‘purer’ members of society to avoid ‘polluting’ vital elements such as air or water.^1 21^ Prior to the use of the term ‘Safai Karamcharis’, manual scavengers were identified by their caste title, such as ‘Methar’, ‘Bhangi’, ‘Balmiki’ or ‘Dalit’. Many of these terms, such as Dalit, which means ‘broken identity’ or ‘broken people’, were used as a derogatory epithet, although anticaste advocates have recently begun to reclaim them.^11 22^ Using these terms, especially to cause insult, was made punishable under Section 3(1)(x) of the Scheduled Castes (SC) and the Scheduled Tribes (ST) (Prevention of Atrocities) Act of 1989, which introduced the labels SC and ST as non-derogatory umbrella terms to refer to these communities.^23^

The deep-rooted cultural and social discrimination against manual scavengers has been exacerbated by a long history of using scavengers to compensate for deficient sanitation infrastructure. During the colonial era, sanitary and public hygiene interventions were frequently piecemeal, lacklustre and concentrated on British residences and cantonment areas.^24 25^ Preconceptions of lower-caste Indians as being ‘savage’ and ‘dirty’ perpetuated negligence of proper sanitation systems in native quarters. This forced local communities to continue to rely on manual scavengers to maintain sanitation, but also exacerbated pre-existing social divides within the new urban order.^26 27^ Residential street waste was managed by either ‘hereditary sweepers’, who were assigned the occupation by birth, or ‘municipal sweepers’, who were government employees but were still predominantly recruited from manual scavenging communities.^28 29^ Occupational hazards were compounded by social discrimination. Scavengers’ work was classified as an essential public service, thereby prohibiting them from taking up other employment or unionising to demand better pay.^26 28^ Beholden to housing supplied by municipal corporations, communities were also often forced to live in the most crowded and unsanitary regions of a city and were frequently subjected to eviction and displacement during urban renewal projects.^30^

Resistance by scavengers, including strikes in Bombay, Delhi and Haridwar, was met with punitive legislation like the Municipal Act of 1900 in Haridwar, which specified that hereditary sweepers could be fined for neglecting to clean private residences or required to forfeit rights to cleaning the building.^29^ In Bombay, the 1890 Municipal Servants Act prevented sanitation workers within the Presidency from leaving their work without 2 months’ notice, punishable by imprisonment, fine or both.^31^ Many urban settlements also tried to pass legislation to municipalise scavenging and to prevent scavengers from earning additional income by selling night soil.^32^

This binary logic of simultaneously acknowledging the need for manual scavenging and disciplining scavenging communities accelerated during the first half of the 20th century. Some states tried to stifle scavenger agency by restricting collective action with laws like the Punjab Municipal Act No. 3 of 1911 or the Uttar Pradesh Act No. 2 of 1916, which remain in place to this day. Meanwhile, leading figures within Congress tried to reconcile nationalist ideals of democratic enfranchisement with an idealised paternalist vision of the caste system.

During the interwar period, Mahatma Gandhi repeatedly described India’s caste system as an organic hierarchy underpinning the smooth running of society in which all factions played an important role and should be respected and developed.^33^ He condemned the practice of untouchability, yet simultaneously glorified the caste-based work of manual scavengers as a ‘sacred’ form of public service—akin to a mother washing dirt off their baby.^34^ Following Gandhi, the essential nature of this service accorded dignity to scavengers but simultaneously denied them the right to strike or challenge their place in society. Although Gandhi’s beliefs evolved, his caste-based idealisation of scavenging led to clashes with the pioneering social reformer and anticaste activist, Dr Bhim Rao Ambedkar, a *dalit* himself.^35^ In contrast to Gandhi, Ambedkar identified scavenging as a forced caste occupation and thereby without choices, resources or dignity.^24^ He claimed that much of Gandhi’s statements were an attempt to use tokenism to appease scavengers to continue their work with minimum resistance.^36^ The dispute came to a head in 1932, when Ambedkar’s demand for a separate electorate for the ‘Depressed Classes’ was granted, and Gandhi announced that he would fast unto death to protest the division of Hindu society.^37^

## Post-Independence

Clashes about the status of scavengers and how to improve their condition continued after Independence. Driven by activist protest and government attempts to gain electoral favour, a series of expert commissions were installed. Resulting reports recommended a mix of technological interventions and social reforms, which were intended to reduce the need for scavenging and turn it into a regulated service with employee protections. Results were mixed.^38 39^

In 1949, the Government of Bombay’s Barve Committee enquired into the living conditions of local scavengers and minimum wages.^40^ At the national level, the first so-called Backward Classes Commission was appointed in 1953 and described the condition of sweepers and scavengers as subhuman while emphasising that mechanical and up-to-date methods of cleaning latrines were needed to end the inhuman practice of doing this work by hand.^40^ Published in 1961, the much-cited Malkani *et al*’s^41^ report recommended implementing technical improvements such as underground drainage, improved latrines and addressing the lack of human dignity at all stages of scavenging. Unfortunately, resulting investment and reform efforts proved insufficient to end demand for manual scavenging and did little to empower communities themselves.

During the late 1960s, the newly formed ‘All India Safai Mazdoor (Cleaning Workers) Congress’ and its various political affiliations launched campaigns for renewed action. Spurred by labour movements and the shift towards emphasising antipoverty measures under the new Congress Leader Indira Gandhi in 1968–69, the National Commission on Labour recommended comprehensive legislation to regulate the working conditions of ‘sweepers and scavengers’ alongside effective enforcement.^40^ Several government schemes also attempted to improve toilet sanitation technology.^40^ However, once again, investment proved too patchy to meaningfully alleviate the need for scavenging. Meanwhile, official enforcement of worker protection proved inadequate. Despite the paternalist language employed by regulators, scavengers continued to work in precarious environmental conditions with little opportunity to escape the cultural and socioeconomic confines of their caste-based profession.

In view of these failings, 1990s Indian governments changed tack by moving away from paternalist measures designed to protect scavengers with legal safeguards to market-based interventions aiming to serve the supply and demand dynamics perpetuating the scavenging sector. In 1993, the Rao administration tried to address both the supply and demand for scavenging labour by outlawing the employment of manual scavengers with the Employment of Manual Scavengers and Construction of Dry Latrines (Prohibition) Act.^42^ Breaking with Independence leaders’ justification of scavenging as a valuable ‘service’, the 1993 Act described manual scavenging as undignified and inhumane work and criminalised the *employment of manual scavengers* rather than manual scavenging itself.^43^ While labour demand was outlawed, labour supply was to be addressed by investment in alternative skilling and trades through the National Scheme for Liberation and Rehabilitation of Scavengers in 1992 and the National Safai Karamcharis Finance and Development Corporation or NSKFDC in 1997.^3^ The Low-Cost Sanitation Scheme, sponsored by the central government, was launched to further reduce demand for scavenging and share the cost of conversion of dry latrines.^2^ Meanwhile, protection of transitioning scavenging communities was to be addressed by the 1994 formation of the National Commission for Safai Karamcharis as a governmental watchdog to investigate all maltreatment of sanitation workers.^42^

Neither illegalisation nor incentivisation succeeded. By targeting employers and failing to address the lack of regular water sources driving ongoing reliance on dry latrines, the 1993 Act did not end demand, but drove it underground. Meanwhile, deep-seated caste and socioeconomic deprivation meant that manual scavengers continued to provide labour to unblock drains and clean latrines, but now lacked legal protection. Cloaked in the language of ‘liberation’ and ‘development’, the new economic schemes failed to address the structural barriers preventing Karamcharis from accessing the reskilling schemes promised by the government. Literacy requirements, bureaucratic hurdles and the need for authorisation by local governments meant that many applications for loans and self-employment projects failed or were never attempted.^1^ In 2020, a public forum acknowledged that allocated funds towards rehabilitation efforts had been underutilised, that the identification of manual scavengers or their families for skill development training was low, and that the impact of funds that had been spent could not be tracked because of poor follow-up.^44^

## NGOs, courts and continuing mandamus

With 1990s reforms stalling, the new millennium saw NGOs and courts emerge as drivers of change. Lawsuits challenged the legal status quo and highlighted how pushing scavenging underground had obfuscated rather than solved problems. Grassroots data gathered among manual scavenging communities on otherwise hidden activities proved vital in both defining the goals of legal action and forcing government authorities to address existing gaps in official data collection.

Beginning in 2002, a coalition of 30 non-profit volunteer organisations led by the group *Jan Sahas* began a campaign to inform and encourage manual scavengers to leave the practice of their own volition with activist and legal support.^11^ Titled the *Rashtriya Garima Abhiyan* (National Dignity Campaign), the organised effort saw approximately 15 000 manual scavenging women boycott their work and enter alternative employment. Parallel surveys significantly improved public understanding of the health and safety risks incurred by manual scavenging communities,^45^ but also highlighted resistance towards allowing local manual scavengers to leave their occupation, with multiple recorded incidents of scavengers being met with verbal and physical abuse, against which constabulary bodies did little.^11^

In 2003, Bezwada Wilson, founder of the non-profit Safai Karamchari Andolan and a member of a manual scavenging caste community, tried to force official recognition of ongoing problems by petitioning the Supreme Court for measures to protect manual scavengers across India.^43^ Lasting for 11 years, the case (Safai Karamchari Andolan and Ors vs Union of India and Ors) was viewed as a ‘Continuing Mandamus’, which meant that the court could be moved to order state and regional authorities to appear, to provide information on current conditions and the status of implementation of previous rulings over a long period of time.^43^ The primary goal of this form of public interest litigation was to pressure central and state governments to both admit to and account for manual scavenging activity occurring within their wards and develop a register of individuals involved in the activity or affected by it.

Court proceedings revealed deeply flawed estimates regarding the total number of manual scavengers. Analysis of the 15th Census of India (2011) indicated that millions of households still had dry latrines with entire communities openly defecating, requiring workers to manually scavenge after them. This number far exceeded decades-old official estimates of between 400,000 and 500,000 scavengers.^42^ Additional findings showed that the government-owned Indian Railways was the single largest national employer of manual scavengers and that the national commission (National Commission for Safai Karamcharis) had only surveyed some districts of India in its national census of Safai Karamcharis.^2 42 46^ The damaging judicial proceedings, surveys and publications resulted in the 2013 passage of the Prohibition of Employment as Manual Scavengers and their Rehabilitation Act (PEMSR), which stipulated the identification of all manual scavengers and dry latrines in India.

Although the PEMSR Act of 2013 has been the backbone of many appeals, institutional reforms and advocacy movements, scavenging and caste-based discrimination persist. According to recent reports, there have been no convictions under the 2013 law.^47^ Activists also complain that acquiring data on the illegal employment of scavengers is often circumvented, as law enforcement and judiciary members are more likely to discriminate against manual scavenging communities than against wealthier and often higher-caste employers.^48^ Meanwhile, PEMSR rehabilitation efforts have also been ineffective due to unclear legal stipulations regarding funding sources, land allocations for housing, etc.^46^

While the mixed outcomes of the PEMSR Act highlight the limits of legal reform alone, the parallel intensification of technocratic initiatives to improve India’s sanitary infrastructure has similarly failed to resolve the challenge of manual scavenging. In 2014, the newly elected government under Narendra Modi announced an ambitious Swachh Bharat Mission (SBM) or Abhiyan (‘Clean India Campaign’) to eradicate open defecation by building approximately 10.4 million toilets for individuals and 50.8 million community toilets nationwide. Toilet designs chosen were either pit latrines, which collect human faeces in a hole in the ground, or pour-flush latrines, which manually or mechanically use water to push faeces to a collection unit.^49^ The SBM also saw the Indian Railways launch an ambitious bio-toilets programme (which biologically treats waste using bacteria) in an attempt to ensure that no human waste is discharged onto railway tracks.^50^ Despite achieving substantial sanitary gains and some success in reducing child mortality,^51^ SBM schemes have, however, mostly sidelined issues related to the rights and protection of manual scavengers. Scavengers remain in demand when toilets fall into disrepair or lack access to sustained water sources and regular maintenance.^52^ The SBM ‘Practitioner’s Guide’ condemned the continuation of manual scavenging but offered no steps for how to eradicate it.^53^ In 2017, the central government reacted to criticism of its sidelining of scavengers by claiming to have tackled the issue by identifying and providing 91% of scavengers with a one-time aid payment of ₹40,000 (roughly equivalent to £485, factoring in inflation)—a claim that was soon disputed after human rights advocates showed that only approximately 7% of the actual population of manual scavengers had been identified.^54^

Given the failure of legislation, technocratic investments and piecemeal executive interventions to resolve the structural barriers faced by scavengers, pressure for further action continues to emanate from Indian NGOs, international organisations and India’s judiciary. In 2019, the World Bank and others issued a critical report on the criminalisation of manual sanitation work.^55^ That year, the Indian Supreme Court, too, questioned the central government on why PPE, such as masks and gas cylinders, which help to prevent asphyxiation in closed sewers, were not provided to sanitation workers, especially those hired to clean manholes. The sitting judge expressed that despite the abolition of untouchability in the constitution 70 years ago, it continued to shamefully perpetuate, expressing that “…In no country in the world, people are sent to gas chambers to die.”^56^

## A state of limbo

As the long list of only partially successful—and occasionally counterproductive—reform efforts shows, it is unlikely that manual scavenging will disappear from India anytime soon. Successive administrations have tried to solve problems with quick fixes like review committees, retraining, outlawing employment and calling for more data. However, there has been little political appetite to address the underlying environmental conditions, entrenched social attitudes, economic precarity and gender biases that have been driving manual scavenging and the marginalisation of scavenging communities. Although it would be wrong to romanticise the paternalist worker protection offered by post-Independence administrations, the increasing neoliberalisation of India’s economy and concomitant focus on labour competition have exacerbated the condition of low-cost, lower-caste workers.^18^

In the absence of an overarching strategy of tackling the identified structural conditions, headlines of scavengers’ deaths spark explosive yet fleeting outrage before returning to the margins of public awareness. Meanwhile, reformers themselves remain divided about the basic question of whether to reformalise or continue to outlaw manual scavenging. In 2020, the judiciary tabled an amendment to the 2013 PEMSR Act, which mandated the development of sanitation technology, while also introducing stricter punishments for offenders.^57^ By contrast, NGOs highlight how bans predominantly harm labourers and not employers by leading to more precarious working conditions.^47^ Instead of bans, some campaigners therefore call for a reformalisation of the scavenging workforce, which would provide legal cover via labour and human rights and—at least theoretically—provide access to health insurance, protective equipment, vaccines and regular health check-ups.^11 55^ By contrast, prominent campaigners like Bezwada Wilson maintain that legalisation would be returning to the paternalistic approach of formalising discriminatory caste-based labour and that manual scavenging needs to be eradicated in its entirety.^1^

In the absence of agreement on how to proceed, scavengers remain stuck in a dangerous state of legal limbo, which has been further exacerbated by the COVID-19 pandemic. Recent reports reveal that the highest percentage of cleaning staff who died of COVID-19 in Delhi consisted of municipal sanitation workers tasked with cleaning streets and handling contaminated material from health centres.^58^ While these data reflect the lack of protection for municipal workers, the extent of the burden on manual scavengers specifically remains difficult to assess due to the ongoing lacuna of data. In addition to the risk posed by infection, the first nationwide lockdown of 2020 meant that many manual scavengers, like the majority of informal workers in India who often migrate from rural to urban areas in search of work in the past,^59 60^ were left with no transport, access to ration houses or food vendors, and daily wages. Without permission to leave their homes, they could not go out looking for work without risking fines, arrests or violent constabulary. Reports of starvation were widespread. Although municipal and contract sanitation workers continued to work during the lockdowns, illegitimate manual scavengers were a part of the resulting mass exodus of the majority informal workforce. Travelling hundreds of kilometres to their hometowns and villages on foot, there were numerous deaths en route from starvation, dehydration, exhaustion, fatal accidents, police brutality, suicide or COVID-19 itself. However, no official death tolls have been released.^61^ Under pressure to find new employment and repay predatory loans, some manual scavengers also shifted to alternate illegal and socially ‘impure’ occupations, such as ‘bone scavenging’ (the illicit sourcing and trading of calcium from the bones of buried corpses).^62^ Far from being over, scavenging thus continues across India.

## Conclusion

This article does not make for light reading—nor is it meant to. Looking back on 120 years of attempts to regulate manual scavenging in India reveals a fundamental tension in addressing the entangled problems of caste-based discrimination and dangerous labour conditions. The interconnection between caste station and impurity has perpetuated an apathy among the public towards manual scavengers and an indifference towards their occupational safety and well-being. Depending on the political *Zeitgeist*, officials employed various strategies to deal with the vicious cycle of demand for manual scavenging due to inadequate sanitary infrastructure and its supply through caste-based labour pools. During the colonial period, British and municipal authorities allowed discriminatory ‘traditional’ practices to continue and expand. Scavenging communities’ attempts to assert agency were met with crackdowns. Following Independence, political acknowledgement of scavengers’ plight sat uneasily with a romanticisation of caste-based notions of Safai Karamcharis’ service to higher castes. A series of paternalist interventions attempted to formalise and regulate work practices not only remained ineffective due to lack of investment and enforcement, but also continued to lock communities into a broader exploitative setting. The 1990s reformers moved away from this paternalistic model of reform and instead followed contemporary neoliberal thinking by attempting to tackle scavenging as an issue of supply and demand. Demand was made illegal, while supply was to be dried out via retraining. What might have been promising policies and schemes, however, tottered due to apathetic enforcement, failure to make support schemes accessible to scavenging communities and the obfuscation of scavengers from official statistics. Since 2013, the most recent wave of legislative reform and technological investment in sanitary infrastructure has gone some way towards reducing the need for scavenging, but has failed to eliminate it or address the entrenched discrimination scavenging communities face.

In many ways, the Swachh Bharat Mission’s official logo of Gandhi’s spectacles perfectly symbolises the ongoing disenfranchisement of scavengers. For over a century, Safai Karamcharis have variously been prosecuted, ‘protected,’ ‘liberated’ or seemingly ‘made redundant’ via technology. However, Karamcharis largely remain spoken for by political elites, yet continue to fight for the agency to speak for themselves.

Regardless of which party leads India over the next decades, manual scavenging and the wider provision of sanitation and sanitary labour need to be seen not as paternalist or purely market-based policy problems, but as collaboration with affected communities requiring an amalgamation of technical, environmental, social and cultural interventions. In view of ongoing demand for sanitary labour, reforms not only need to end exploitative informal employment of scavengers—especially by public bodies—but also address the desires and needs of the broader sanitation worker communities for accessible, safe and dignified housing and working conditions, as well as affordable healthcare, insurance, employee benefits and children’s education. A nationwide census and record of all manual scavengers as well as surveys of scavengers’ aspirations alongside proper representation in decision-making bodies are important first steps towards achieving a more participatory form of engagement.

## Data Availability

All data relevant to the study are included in the article.

## References

[1] Joshi D, Ferron S. Manual scavenging - a life of dignity? Waterlines 2007;26:24–7. 10.3362/0262-8104.2007.054

[2] National Commission for Safai Karamcharis (NCSK). Annual report 2012-13. New Delhi: Ministry of Social Justice and Empowerment, Government of India, 2014.

[3] National Safai Karamcharis Finance & Development Corporation (NSKFDC). FAQ, 2021. Available: https://nskfdc.nic.in/en/content/home/faq [Accessed 18 Dec 2020].

[4] Mander H. Resource handbook for ending manual scavenging. New Delhi, India: International Labour Organisation, 2014.

[5] PTI. 631 people died cleaning sewers, septic tanks in last 10 years: NCSK. Deccan Herald, 2020. Available: https://www.deccanherald.com/national/631-people-died-cleaning-sewers-septic-tanks-in-last-10-years-ncsk-890242.html [Accessed 18 Dec 2020].

[6] Tiwari RR. Occupational health hazards in sewage and sanitary workers. Indian J Occup Environ Med 2008;12:112. 10.4103/0019-5278.44691 20040968 PMC2796749

[7] Dalberg Advisors. Sanitation worker safety and livelihoods in India: a blueprint for action—phase 1: understanding the problem. New Delhi, India, 2017.

[8] Salve P, Bansod DW, Kadlak H. Safai karamcharis in a vicious cycle: a study in the perspective of caste. Economic & Political Weekly 2017;52:37–41 https://www.epw.in/journal/2017/13/perspectives/safai-karamcharis-avicious-cycle.html

[9] People’s Union for Civil Liberties- Karnataka (PUCL-K). A millennial struggle for dignity: manual scavenging in Karnataka. Bengaluru: PUCL-K, 20192019.

[10] Jan Sahas Social Development Society. Socio economic status of women manual scavengers: baseline study report. Jan Sahas Social Development Society 2014;2014.

[11] Human Rights Watch (HRW). Cleaning human waste: manual scavenging, caste,and discrimination in India. United States of America, 2014.

[12] Prasad SC, Ray I. ‘When you start doing this work, it is hard to eat dal’, life and work of manual scavengers. Economic and Political Weekly 2017;53:25–7 https://www.epw.in/journal/2018/32/commentary/%E2%80%98when-you-start-doing-work-it-hard-eat.html

[13] Rangamani S, Obalesha KB, Gaitonde R. Health issues of sanitation workers in a town in Karnataka: findings from a lay health-monitoring study. Natl Med J India 2015;28:70–3. 26612148

[14] WaterAid. Safety and wellbeing of sanitation workers during COVID-19 in South Asia, 2020. Available: https://washmatters.wateraid.org/sites/g/files/jkxoof256/files/safety-and-wellbeing-of-sanitation-workers-during-covid-19-in-south-asia-synthesis-report.pdf

[15] Sunderland EM, Hu XC, Dassuncao C, et al. A review of the pathways of human exposure to poly- and perfluoroalkyl substances (PFASs) and present understanding of health effects. J Expo Sci Environ Epidemiol 2019;29:131–47. 10.1038/s41370-018-0094-1 30470793 PMC6380916

[16] Ambedkar BR. Annihilation of caste: Dr. Babasaheb Ambedkar writings and speeches. Compiled by Vasant Moon. Mumbai: Education Department, Government of Maharashtra, 1936.

[17] Madan TN. Caste, social differentiation. Encyclopedia Britannica, 2019. Available: https://www.britannica.com/topic/caste-social-differentiation [Accessed 3 Jun 2022].

[18] Dubey SY, Murphy JW. Manual scavenging in Mumbai: the systems of Oppression. Humanity Soc 2021;45:533–55. 10.1177/0160597620964760

[19] Senart É, Ross ED. Caste in India: the facts and the system. London: Routledge, 1930.

[20] Douglas M. Purity & danger. 6th edn. New York: Routledge & Kegan Paul Ltd, 1966.

[21] Sharma M. Caste and nature: dalits and Indian environmental politics. New Delhi: Oxford University Press, 2018.

[22] Dey S. Manual scavenging: a national scourge. TerraGreen 2019;10.

[23] Manasi S, Babu MU. Small conflict uncovers big issues - conflict between the manual scavenging community and savanur town. Conflicts around drinking water and sanitation in india. Forum for policy dialogue on water conflicts in India. Pune, Maharashtra, 2014.

[24] Guru G. Archaeology of untouchability. Economic & Political Weekly 2009;44 https://www.epw.in/journal/2009/37/special-articles/archaeology-untouchability.html

[25] Bashford A. Imperial hygiene: a critical history of colonialism, nationalism and public health. Basingstoke: Palgrave Macmillan, 2004.

[26] Prashad V. Untouchable freedom: a social history of a Dalit community. Delhi: Oxford University Press, 2000.

[27] Kidambi P. Housing the poor in a colonial City: the Bombay improvement trust, 1898-1918. Stud Hist 2001;17:57–79. 10.1177/025764300101700103 18326108

[28] Mandal S. Through the lens of pollution: manual scavenging and the legal discourse. Voice of Dalit 2008;1:91–102. 10.1177/0974354520080107

[29] Khalid A. ‘Unscientific and insanitary’: hereditary sweepers and customary rights in the United Provinces. In: Johnson R, Khalid A, eds. Public health in the British Empire: intermediates, Subordinates, and the practice of public health, 1850-1960. London: Routledge, 2012: 67–89.

[30] Thatra G. Dalit Chembur: Spatializing the caste question in Bombay, C. 1920s-1970s. Journal of Urban History 2020:1–35.

[31] Masselos J. Jobs and jobbery: the sweeper in Bombay under the raj. Indian Eco Soc History Rev 1982;19:101–39. 10.1177/001946468201900201

[32] Aloysius G. Nationalism without a nation in India. New Delhi, India: Oxford University Press, 2011.

[33] Gandhi MK. The collected works of Mahatma Gandhi, 98 Volumes (Electronic Book). Publications Division Government of India, New Delhi. Vol 21: 21 July- 21 Nov 2020, 1924. Available: https://www.gandhiashramsevagram.org/gandhi-literature/mahatma-gandhi-collected-works-volume-21.pdf

[34] Gandhi MK. The ideal Bhangi. Harijan 1936;42 http://www.columbia.edu/itc/mealac/pritchett/00ambedkar/timeline/graphics/txt_gandhi_1936_bhangis.pdf

[35] Barua A. Revisiting the Gandhi–Ambedkar debates over ‘Caste’: the multiple resonances of varņa. J Human Value 2019;25:25–40. 10.1177/0971685818805328

[36] Ambedkar BR. What Congress and Gandhi have done to the untouchables. 2nd edn. Thacker, 1946. https://www.mea.gov.in/Images/attach/amb/Volume_09.pdf

[37] Akhilesh P. Do sanitation workers identify themselves with Ambedkar and Gandhi? 2021. Available: https://theleaflet.in/do-sanitation-workers-identify-themselves-with-ambedkar-and-gandhi/#:~:text=from%20this%20land.-,Sanitation%20workers%20see%20Ambedkar%20as%20a%20doctor%20who%20not%20only,Gandhi%20together%20in%20their%20home [Accessed 25 May 2022].

[38] Srinivas L. Master-servant relationship in a cross-cultural perspective. Economic & Political Weekly 1995;30.

[39] Ministry of Home Affairs. Report of the Committee on customary right to scavenging. New Delhi: Government of India, 1966.

[40] Srivastava BN. Manual scavenging in India. New Delhi: published for Sulabh international social service organisation by concept publication 1997.

[41] Malkani NR, Rajabhoj PN, Balmiki KL. Report of the scavenging conditions enquiry Committee. Delhi: Manager of Publications, 1961. https://indianculture.gov.in/report-scavenging-conditions-enquiry-committee-0

[42] National Commission for Safai Karamcharis (NCSK). Annual report 1994-95. New Delhi: Ministry of Social Justice and Empowerment, Government of India, 1995.

[43] Mehta A, Bhandari N. Manual scavenging: an endless cycle of false promises & failed policies. Law School Policy Review & Kautilya Society 2020 https://lawschoolpolicyreview.com/2020/04/13/manual-scavenging-an-endless-cycle-of-false-promises-failed-policies/

[44] Mitra R. No funds released for rehabilitation of manual scavengers, earlier funds Unutilized: the new Indian express, 2020. Available: https://www.newindianexpress.com/nation/2020/sep/21/no-funds-released-for-rehabilitation-of-manual-scavengers-earlier-funds-unutilized-govt-2199858.html [Accessed 28 Nov 2020].

[45] Jan Sahas Social Development Society. Socio economic status of women manual scavengers: baseline study report 2014.

[46] Jitendra. Manual scavenging prohibition bill: how effective? 2013. Available: https://www.downtoearth.org.in/news/manual-scavenging-prohibition-bill-how-effective-42151 [Accessed 18 Jan 2021].

[47] Thekaekara MM. Why the proposed manual scavenging prohibition bill looks good only on paper, 2020. Available: https://thewire.in/caste/manual-scavenging-prohibition-bill-2020-caste-rehabilitation [Accessed 28 Dec 2020].

[48] Grover P. Despite a slew of policies, why manual scavenging is still a problem for India in 2018, 2018. Available: https://theprint.in/india/governance/why-manual-scavenging-is-still-a-problem-for-india-in-2018-despite-a-slew-of-policies/127289/ [Accessed 21 Dec 2020].

[49] Ministry of Drinking Water and Sanitation (MoDWS) GoI, WASH Institute, Bill & Melinda Gates Foundation (BMFG), and Kantar Public. Review of health data in selected ODF and Non-ODF districts under the SBM (study report), 2017. Available: https://jalshakti-ddws.gov.in/sites/default/files/BMGF_Health_Impact_Study_final.pdf

[50] Khan Y. Indian railways installs aircraft-style toilets in all long-distance train, 2021. Available: https://www.moneycontrol.com/news/trends/indian-railways-installs-aircraft-style-toilets-in-all-long-distance-train-7003191.html [Accessed 8 Jan 2022].

[51] Kishor D. Role of sanitation workers in Swachh Bharat. Int J Human Soc Sci Stud 2017;2017:271–81.

[52] Sagar. Down the drain: how the swachh Bharat mission is heading for failure, 2017. Available: https://caravanmagazine.in/reportage/swachh-bharat-mission-heading-failure [Accessed 28 Dec 2020].

[53] Rohilla SK, Luthra B, Bhatnagar A. Septage management: a practitioner’s guide. New Delhi: Centre for Science and Environment, 2017.

[54] Yadavar S. Government claims to have assisted 91% of India’s manual scavengers, without counting 93% of them, 2017. Available: https://scroll.in/article/841580/government-claims-to-have-assisted-91-of-indias-manual-scavengers-without-counting-93-of-them [Accessed 27 Nov 2020].

[55] World Bank, ILO, Water Aid and WHO. Health, safety and dignity of sanitation workers: an initial assessment. Washington, DC: International Bank for Reconstruction and Development / The World Bank, 2019.

[56] PTI. Nowhere in the world people sent to gas chambers to die, says SC on manual scavenging, 2019. Available: https://www.indiatoday.in/india/story/nowhere-in-the-world-people-sent-to-gas-chambers-to-die-says-sc-on-manual-scavenging-1600455-2019-09-18 [Accessed 18 Dec 2020].

[57] Akhilesh P. Banning manual scavenging in India: a long, complex passage, 2020. Available: https://www.downtoearth.org.in/blog/rural-water-and-sanitation/banning-manual-scavenging-in-india-a-long-complex-passage-73441 [Accessed 24 Nov 2021].

[58] Rajput A. Delhi: half of Covid dead under municipal corporations are safai karamcharis, 2021. Available: https://indianexpress.com/article/cities/delhi/delhi-half-of-covid-dead-under-municipal-corporations-are-safai-karamcharis-7333365/ [Accessed 12 Aug 2021].

[59] Sinha C, Kumar M. Conceal or not? Management of Dehumanized work identity among lower caste domestic workers and Non-domestic scavenging workers. South Asian J Human Res Manag 2018;5:173–93. 10.1177/2322093718787097

[60] Lenka AK. Health, safety and well-being of workers in the informal sector in India. In: Lessons for emerging economies; health, work, and state response to person engaged in sanitation work: some issues and challenges. Singapore: Springer Nature Singapore Pte Ltd, 2019: 215–31.

[61] Damini N. Govt. has no data of migrant workers death, loss of job, 2020. Available: https://www.thehindu.com/news/national/govt-has-no-data-of-migrant-workers-death-loss-of-job/article32600637.ece [Accessed 21 Dec 2020].

[62] Akhilesh P, Balmini A, Balmiki S. Lockdown effect: manual scavengers turn to selling human bones simply to survive, 2022. Available: https://thewire.in/rights/lockdown-effect-manual-scavengers-turn-to-selling-human-bones-simply-to-survive [Accessed 28 May 2022].

